# The journey of adolescent paranoia: A qualitative study with patients attending child and adolescent mental health services

**DOI:** 10.1111/papt.12385

**Published:** 2022-02-12

**Authors:** Jessica C. Bird, Daniel Freeman, Felicity Waite

**Affiliations:** ^1^ Department of Psychiatry University of Oxford Oxford UK; ^2^ Oxford Health NHS Foundation Trust Oxford UK; ^3^ South London and Maudsley NHS Foundation Trust London UK

**Keywords:** early intervention, interpretative phenomenological analysis, psychosis, psychotic experiences, youth mental health

## Abstract

**Objectives:**

Paranoia is most likely to emerge in adolescence. In adolescents with mental health disorders, the disruptive effect of paranoia on social relationships could worsen outcomes. However, little is known about clinical presentations of paranoia at this age. We therefore explored the development, experience, and impact of paranoia in adolescent patients.

**Design:**

A qualitative interview design with interpretative phenomenological analysis was used.

**Method:**

Twelve adolescents (11–17 years) with paranoia attending child and adolescent mental health services were interviewed.

**Results:**

Adolescents described a journey starting with their awareness of paranoia beginning to a paranoid experience of mistrust and fear of others, and, subsequently, their adjustment to paranoia in daily life. Paranoia onset was rooted in the discovery of interpersonal threat and personal vulnerability, shaped by challenging peer interactions, becoming aware of danger in the world, and personal adverse experiences. The paranoia experience included a struggle to trust friends, anticipating threat with intense fear, and using defensive strategies to keep safe. Adolescents described how the paranoia experience was confusing, negatively impacted self‐concept, held them back from teenage life, and caused disconnection from friends. Longer‐term responses to paranoia reflected a tension between reluctantly resigning to the experience and trying to resist the impact.

**Conclusions:**

The journey of paranoia in adolescence involves navigating multiple tensions, with young people balancing independence with vulnerability, trust with mistrust, and the desire to socialise with a fear of danger and deception. Decisions about how to respond to paranoia are likely to determine the next stage of their journey.

**Practitioner points**

- Recent evidence suggests paranoia may be a common and clinically important problem in patients attending child and adolescent mental health services (CAMHS).
- During qualitative interviews, twelve adolescent patients described distressing paranoid concerns that had a clear impact on their mental health and social relationships but were broadly overlooked by services and untreated.
- The findings provide a novel developmental framework of paranoia in adolescence as an understandable response to growing up in an often‐threatening social world. For adolescent girls, this may also include discovering the realities of sexual harm in their daily lives.
- The journey of paranoia described in this study could be used by clinicians to frame conversations with young people about their paranoia and inform the development of effective interventions.

## BACKGROUND

Paranoia – the inaccurate idea that others intend to cause harm – is likely to first emerge in adolescence. Starting at the onset of puberty, adolescence is a formative period of neurobiological and socio‐developmental change that ends with the achievement of independent adult roles (Sawyer et al., 2018). This transition from childhood to adulthood is a vulnerable period for the development of mental health problems including psychotic experiences (Kessler et al., 2007; McGrath et al., 2016). It is the age when paranoid thoughts may be most prevalent (Bird et al., 2019; Freeman et al., 2011), and recent evidence suggests paranoia is a common presentation in those attending child and adolescent mental health services (CAMHS) (Bird et al., 2021). Yet over time, these emerging suspicions will take different paths, likely diminishing for many adolescents but persisting in others. For those with worsening paranoia, expecting harm from others is likely to be corrosive for peer relationships and mental health (Bird et al., 2021). Identifying and treating persistent paranoia in adolescents accessing CAMHS is therefore important.

Clinical approaches to paranoia in youth may require a tailored approach that acknowledges the unique life‐stage issues associated with adolescence (McGorry, 2007) and potential differences in phenomenology as paranoia first emerges (McGorry & Nelson, 2016). So far, however, a detailed insight into clinical presentations of paranoia at this age is lacking, and there have been no qualitative studies of paranoia in adolescents. We therefore explored the lived experience of paranoia in adolescents attending CAMHS, focusing on two broad aspects of the experience. The first aspect was the phenomenology of paranoia and its clinical presentation in these patients. The second aspect was adolescents’ understanding of the development of paranoia and its impact in their daily lives. This focus on meaning‐making was guided by the authors’ interest in developing cognitive behavioural therapies for paranoia and was addressed using interpretative phenomenological analysis (IPA) (Smith, 1996) – a qualitative approach specifically designed for understanding how individuals make sense of lived experience.

## METHOD

### Design

A qualitative study using IPA was conducted. The study received approval by an NHS Research Ethics Committee (Ref: 17/SC/0539).

### Participants

Purposive homogenous sampling was used to recruit adolescent patients with paranoia. Inclusion criteria were adolescents: aged 11–17 years; attending CAMHS; and reporting elevated paranoia, defined as 23+ on the Bird Checklist of Adolescent Paranoia (B‐CAP) (Bird et al., 2020). Exclusion criteria were: a diagnosis of psychosis/suspected psychosis; a moderate/severe learning disability; or an inability to undertake an interview in English.

Participants were identified following their participation in a quantitative study examining paranoia in CAMHS (Bird et al., 2021), with high scorers considered potentially eligible. Of the 17 adolescents screened, 3 declined to participate and 2 were unsuitable as they were not currently experiencing paranoia. The remaining 12 adolescents were eligible and provided either assent with parental consent (11–15 years) or individual consent (16–17 years). The interviewer had no pre‐existing relationships with any participant.

### Procedure

Individual interviews were conducted by JCB in either a mental health clinic (*n *= 9), the adolescent's home (*n *= 2), or a university building (*n *= 1). A copy of the interview schedule and details about its development are provided in the Supplementary materials. Interviews, which lasted 30.3–84.5 min (mean = 64.5, *SD* = 14.6), were audio‐recorded and transcribed verbatim. Transcripts were anonymised and participants were assigned pseudonyms. Participants completed a form about their demographic details, their view of why they attended CAMHS, whether they were receiving support for paranoia, and if they wanted help for it. Participants were not invited to provide feedback on the transcripts due to feasibility constraints.

### Analysis

Interpretative phenomenological analysis (IPA) was led by JCB and followed the procedure by Smith et al. (2009) First, each transcript was read and re‐read to familiarise and immerse the self in the data. At this stage exploratory notes including descriptive, linguistic, and conceptual comments were made, with a focus on what mattered to the participant and the meaning of their experiences. Next, emergent themes were drawn from the exploratory comments, synergising idiosyncratic features with conceptual interpretations. Connections between these themes were then explored and clustered into an individual structure. This process was repeated for all twelve accounts, bracketing ideas from the previous interview to ensure an idiographic understanding of each participant's experience. Patterns of similarity and difference across the individual analyses were then identified and organised into an overall structure of superordinate and subordinate themes. These themes were then refined by re‐examining each participant's interview to explore nuances in meaning and ensuring the final interpretation was grounded in young people's words.

Yardley's (2008) criteria for demonstrating credibility in qualitative research, adapted for IPA (Smith et al., 2009), were used throughout the study. Regular supervision with FW and DF was also used to triangulate perspectives, perform credibility checks, and collaboratively refine the developing interpretation. Full details of the steps taken to demonstrate the Yardley criteria, and a description of how reflexivity, supervision, and independent ratings were used to enhance validity, are in the supplementary materials.

## RESULTS

Participant characteristics are shown in Table 1. All participants were White British; eleven were attending outpatient CAMHS and one was an inpatient. Participants presented to services primarily with affective symptoms and self‐harm/suicidality, whilst two participants also had diagnoses of autism.

**TABLE 1:** Participant characteristics

Name^a^	Age	Gender	Presenting problems	Paranoia score	Offered help	Want help
Katie	16	F	Depression, self‐harm, suicidality, emotion dysregulation	63	No	Yes
Megan	16	F	Depression and anxiety	41	No	Yes
Jack	11	M	Generalised anxiety	57	Unsure	Yes
Ashley	12	F	Autism, anxiety, depression, self‐harm, poor sleep	69	Unsure	Yes
Nathan	15	M	Anxiety, depression, suicidality, emotion dysregulation	59	No	Yes
Chloe	14	F	Self‐harm, low mood, anxiety, emotion dysregulation	29	No	Yes
Emily	16	F	PTSD, anxiety	47	Unsure	Yes
Sophie	16	F	Self‐harm, suicidality, eating difficulties	38	Yes	Unsure
Holly	15	F	Depression, emotion dysregulation, self‐harm, suicidality	59	No	Unsure
Sam	12	M	Autism, emotion dysregulation, anger	58	No	Yes
Olivia	16	F	Depression, emotion dysregulation, suicidality, anxiety	55	No	Unsure
Lucy	17	F	Anorexia nervosa, anxiety, self‐harm, suicidality	57	No	Yes

^a^ Pseudonyms used to maintain anonymity.

**FIGURE 1:**
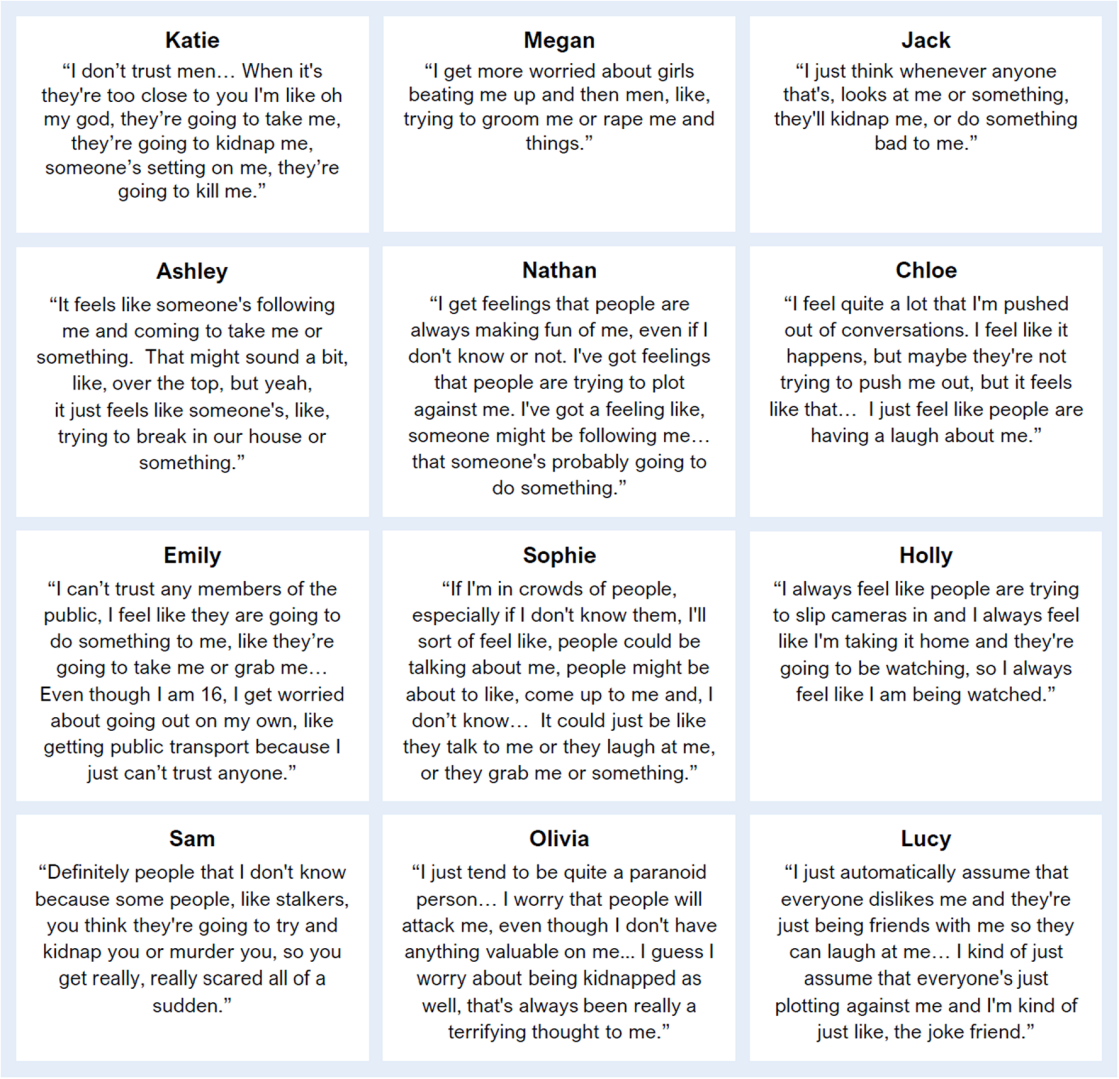
Example of young peoples’ paranoid concerns

Participants described a variety of paranoid concerns, including deliberate social (e.g., secretly made fun of), emotional (e.g., upset on purpose), sexual (e.g., raped), and physical harm (e.g., beat up or killed) (Figure 1). All twelve participants described fears of physical harm from others, which predominantly focused on strangers, and, for ten participants, included a fear of kidnap. Five of nine female participants expressed fears of sexual assault from men. Six adolescents also described suspicions about peers, with concerns of social/emotional harm in situations where this was unlikely. Three participants reported paranoia on social media.

The prominence of paranoia within the adolescents’ broader clinical presentations varied, but none had paranoia recorded as a presenting problem in clinical notes. Five participants understood mistrust/paranoia as central to the reasons they attended CAMHS, although most framed it differently (e.g., anxiety, relationship problems). The remaining seven understood paranoia as an additional problem to the focus of CAMHS interventions; only one of whom had discussed paranoia with a CAMHS professional. Most participants had not been offered support for paranoia despite most saying they wanted it (Table 1).

**FIGURE 2:**
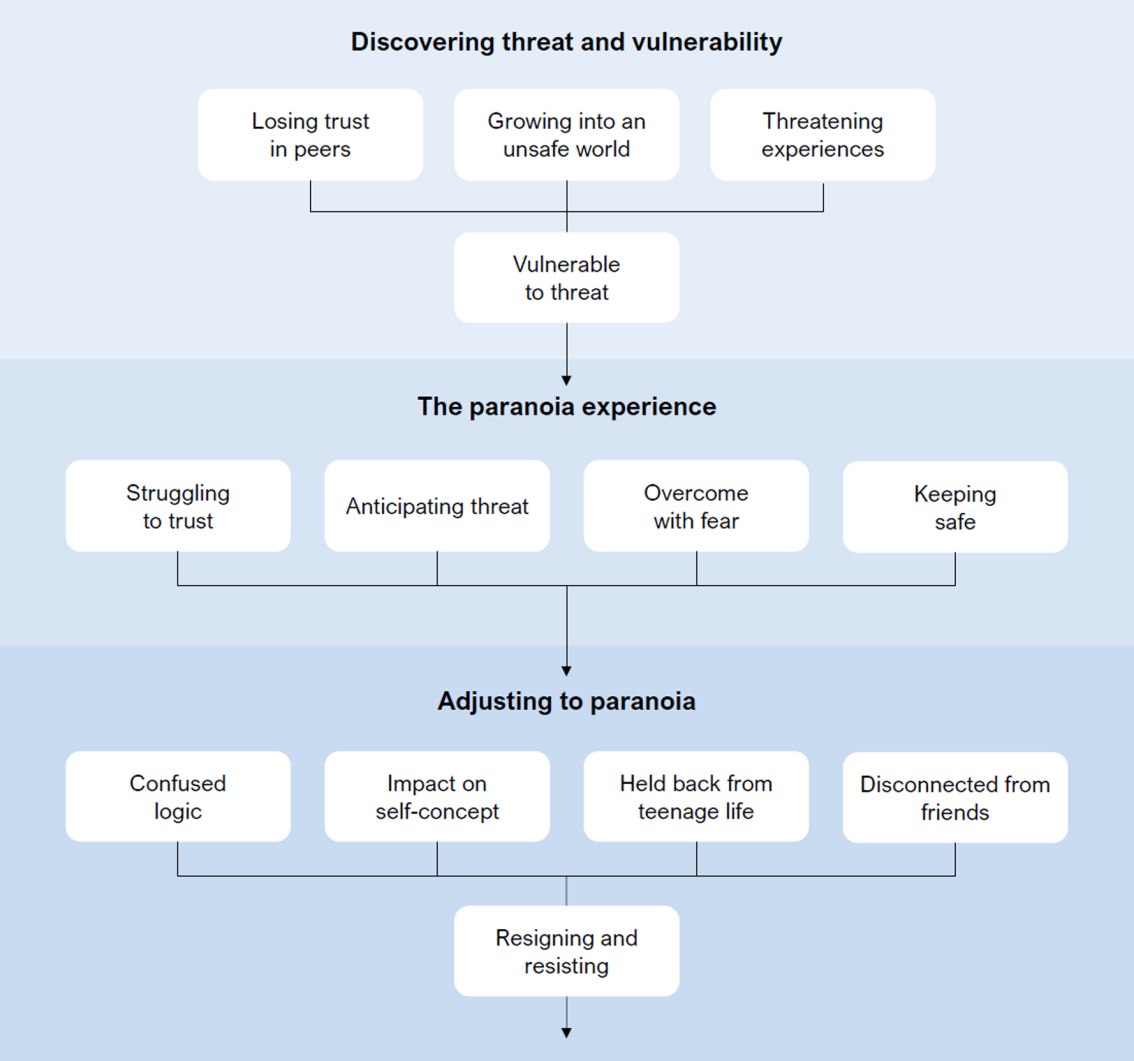
Visual representation of the journey of adolescent paranoia

The IPA analysis described a three‐stage journey of adolescent paranoia, each represented by a super‐ordinate theme (Figure 2). These stages described adolescents’ understanding of how paranoia developed, the core features of their paranoia, and, finally, ongoing attempts to make sense of the experience and its impact. Participant contributions and additional quotes for each theme are in the Supplementary Tables S2–S6.

### Theme 1: Discovering threat and vulnerability

Central to the onset of paranoia was a process of discovering threat and vulnerability. First, all participants described learning more about the possibility of threat from others via three experiences: ‘losing trust in peers’, ‘growing into an unsafe world’, and ‘threatening experiences.’ The discovery of threat through these experiences contributed to a core sense of personal vulnerability to interpersonal harm, from which paranoia emerged.

#### Losing trust in peers

I had a best friend who, like, I definitely can’t trust now, and we were really close, and she just turned on me and turned all my other friends against me for no reason… it was horrible and that made me not really want to trust anyone else. ‐ Emily

For most participants, challenging peer interactions contributed to a shift in assumptions about trust. This included changeable friendships, gossip, rumours, subtle exclusion, and bullying. These experiences contributed to a growing realisation that people, and especially teenagers, were untrustworthy. Many described how paranoia emerged from significant experiences of betrayal within peer relationships. The process of losing trust in peers was often gradual, but some recalled key incidents of falling out with peers that, although typical for adolescence, substantially altered their beliefs about others and underpinned paranoia onset.

#### Growing into an unsafe world

When you always hear things on the news and there's so much of it going on nowadays, and your mother's always warning you and your grandparents are always warning you, it's just so difficult to not have those thoughts. Especially as I’m female, I'm not being sexist, but they're more targeted for assault and stuff. It's just got worse over the years. – Chloe

Six participants described how paranoia emerged in the context of growing up: they were becoming more aware of danger in the world whilst also becoming more exposed to these dangers. This often occurred as they started to go out alone, with four participants saying this was when their paranoia began. The reality of interpersonal dangers such as murder, rape, and terrorism was highlighted to adolescents from the news, warnings from adults, and anecdotes from friends and family. Becoming more aware of danger could lead adolescents to re‐evaluate their childhood assumptions of safety, contributing to an increasing view that the world was not safe. Gender was important in the awareness of threat, with several girls describing a realisation that sexual assault is a risk they face in society. Yet as participants were becoming more aware and exposed to potential danger, they didn't yet know how to protect themselves. The prospect of facing these dangers alone therefore seemed frightening:

You're kind of like, I've got the world to grow into, but it's not something safe. It's something I've got to always be careful of because I'm not going to be able to protect myself… It's a lot more scary because you know you're growing up and you're going to be facing things more independently, [but] you don't really know how to deal with it ‐ Sophie

#### Threatening experiences

Probably after I got groomed, things started to come. And then when I got cheated on, I just thought everyone. And then after I got beat up, I just think all strangers… even though the police said, you were just in the wrong place at the wrong time, I always think, no, it's everyone now – Megan

Eight participants identified threatening experiences that contributed to paranoia onset. These included explicit trauma (i.e. sexual assault/physical assault) and subjectively frightening experiences such as being approached by strangers, ‘cat‐calling’ on the street, and unsettling anomalous experiences. Throughout the accounts, participants described how their concerns had generalised from these frightening experiences. This process was gradual for a number, with an accumulation of experiences generalising their sense of threat over time. Others described how individual incidents led to a dramatic shift in perspective, including a realisation that dangers could happen to them. This generalisation was also influenced by ‘close calls’ that drew attention to harm that could have occurred.

#### Vulnerable to threat

The sense of vulnerability underpinning paranoia included three subthemes: ‘uncertainty about others’, ‘self as a target’, and ‘defenceless and unprotected’.

##### Uncertainty about others

You just don't know when it's going to happen and that always scared me, because a lot of things in my life, when I got mugged, I never thought that would happen to me ever, and then it did… So it makes me think, what else? Obviously, anything can happen, so it's just made it more of a worry in my head. *–* Lucy

Through their experiences, participants described becoming increasingly aware that people are not always as they seem, and that misplaced trust can have harmful consequences. Experiences of broken trust and direct harm had brought significant doubt into their judgements about others, including friends, and, as a result, many described feeling uncertain in decisions about trust. There was significant concern that threat from others was unpredictable and could happen at any time, with this uncertainty contributing to the generalisation of paranoia from threatening experiences. The uncertain nature of threat left adolescents feeling more at risk, prompting a perceived need for constant vigilance.

##### Self as a target

I sort of feel like people will look at me like they know; they know I'm a bit weird and they know that I'm a bit different and it feels like people will pick up on that, and sort of find me an easier target. – Olivia

Participants described how paranoia often emerged from a view of themselves as a target, often linked to negative perceptions of themselves in relation to others. For example, some participants’ beliefs that they were unlikable made it seem more likely that others wanted to harm them and created doubt about whether friendliness was genuine. Feeling like a target was also linked to appraisals that harm is deserved and, as a result, likely to be an ongoing threat. Gender was an important factor in girls’ view of themselves as a target, and notably, both Megan and Lucy described how unwanted male attention made their appearance as ‘pretty’ girls a source of threat.

##### Defenceless and unprotected

I think it can be the fact I'm very weak. I really am not strong… So I think that can make me feel a lot worse because it's like, if something did happen I'm not strong enough to defend myself or get away, and I can easily be overpowered. – Holly

Paranoia was also rooted in the adolescents’ views of themselves as defenceless and unprotected, and thus more likely to face severe consequences if harmed (Raihani & Bell, 2019). For concerns of physical harm this was often linked to beliefs about the self as weak or a distrust in others to protect them. For example, Katie described feeling unsafe in public with friends because “if someone, like, touched me or took me, I don't think I trust [my friends] to get me back.” Feeling unprotected could also be influenced by insecure parental attachments (Bowlby, 1973), with Ashley reflecting that abandonment fears made kidnap more frightening. For social concerns, defencelessness was linked to broader social vulnerabilities where difficulties fitting in meant the risk of friends turning on them was high. This was evident for Sam: “I've only got two or three friends, so I'm really scared of my friends because at one point I might be at zero so I’ll be alone.”

### Theme 2: The paranoia experience

Experiences of paranoia comprised four core components: ‘struggling to trust’, ‘anticipating threat’, ‘overcome with fear’, and ‘keeping safe.’

#### Struggling to trust

When you do have trust for someone, well, really it's just one of the best things you can have; it's just trust. But the opposite, when you don't have trust for someone, especially if you’re close to them… is just a horrible feeling, because you've known them for so long, and you feel like you can't talk to them about stuff you should be able to. – Nathan

Participants described finding it incredibly hard to trust, with many explaining it took a long time, and, even then, several could not “ever really trust anyone 100 percent” (Emily). Trust was typically viewed as fragile with an assumption that others will ultimately prove untrustworthy, and, so, trusting made them vulnerable to getting hurt. Decisions about trust were therefore not to be taken lightly ‐ most participants described trust as highly important and a number thought they took it more seriously than their peers. It was also viewed as necessary and something the adolescents explicitly longed for in friendships, as having trust in others was said to bring a sense of happiness and allowed them to relax, be themselves, and feel safe. Yet despite their desire for trust, adolescents described a tendency towards mistrust in their relationships, remaining wary of others and frequently doubting people's intentions.

#### Anticipating threat

Even at home where everything you think is safe, you still have to look out for people, because people can, like, get through your keyhole. They might know your number for your key lock or they can pick locks, so I still get really, really scared. So when you see somebody out near your path, looking straight at you through your windows, you automatically think ‘hide’. – Sam

All participants described a dominant cognitive mode of anticipating threat from others (Freeman, 2016). This included being on the lookout for danger in social situations, excessively focusing on the actions of others, picking up subtle social cues, and over‐analysing what people might do. These processes contributed to self‐consciousness in social situations, with several adolescents over‐thinking both how they come across and how others react to them. Young people frequently felt on edge around others, with paranoid concerns often described as a ‘feeling’ of threat. Participants also described a tendency to interpret ambiguous interactions as hostile, jump to threatening conclusions, and worrying about the worst‐case possibilities of what could happen. The anticipation of threat was frequently experienced as something always present in young peoples’ minds, which some described as “a constant state of worry” (Holly).

#### Overcome with fear

Sometimes I can take my mind off of it, if my Mum, like, speaks with me, but then sometimes it just doesn't work. Sometimes I get, like, proper anxious, I try and breathe, I just get, I just get so anxious of people staring at me. *–* Jack

Many adolescents described strong emotional responses to paranoid thoughts. These responses, which included panic, terror, and anger, were often experienced as sudden, intense, and overwhelming. For three participants the fear made it hard to sleep. Intense emotions often made it difficult for adolescents to evaluate objectively paranoid thoughts, with several recalling increased conviction in moments of panic. The strong emotions often led to feelings of being out of control, and two participants recalled self‐harm or suicidal threats in response to paranoia. Several adolescents expressed feeling powerless to the emotions evoked and struggling to use coping strategies to calm down. A number also reflected how existing low mood, anxiety, and stress made strong reactions to paranoid thoughts more likely. Conversely, two participants discussed how pre‐existing suicidality and low mood could reduce an initial panic, because as Sophie explained, “if they do attack me, what do I care, really?”

#### Keeping safe

I prefer being inside or where I live because no‐one knows where I live… I don't let anyone know, apart from my really close mates… It's like there's no way that someone can get me from outside or from inside if they don't know where I live – Katie

In response to paranoid concerns participants used a range of defensive strategies to keep themselves safe (Freeman et al., 2007). This included avoiding certain situations, particularly those involving many people, or not staying out for too long. When they were outside, participants tried to minimise risk by keeping a low profile, for example, avoiding eye contact, putting their head down, not talking to strangers, and keeping a distance from crowds. For Lucy, this included wearing baggy clothes to avoid sexual attention from men. Participants took further precautions in public including keeping their back to a wall, having a clear view of the room, planning escape routes, and being ready to defend themselves. Many also described attempts to escape, either by walking faster or running to a place of safety. Several participants further relied on others to protect them, for example, by only going out with trusted people. Six adolescents described defences against social threats, such as keeping others at a distance, not sharing personal details that could be used against them, and generally withdrawing from social interactions.

### Theme 3: Adjusting to paranoia

There was an ongoing process of adjusting to the experience and impact of paranoia including five subordinate themes: ‘confused logic’, ‘impact on self‐concept’, ‘held back from teenage life’, ‘disconnected from friends’, and, finally, ‘resigning and resisting’.

#### Confused logic

That feeling of absolute terror, like all logic will fly out the window. It's like, ‘oh, random person on the street – murderer’. It's crazy how your mind can just sort of flip things… I just go, oh, come on, you're being stupid. And then my mind's like, no, no, absolutely not. – Olivia

Most adolescents described a sense of confused logic about paranoid thoughts, arising from an awareness their concerns were most likely unfounded and excessive. A number explicitly referred to paranoid thoughts as ‘irrational’ or ‘delusional’, and several described their mistrustfulness as ‘unhealthy’. This was less apparent for the youngest three participants (11–12 years old), although they all still described questioning to some extent the accuracy of their fears. For example, Ashley (12y) said her fears would be seen by others as “over the top” as they did not “sound real.” The awareness that fears were unfounded often created a sense of dissonance, confusion, and distress, with several adolescents expressing difficulty understanding why they felt and acted this way.

#### Impact on self‐concept

I don't like myself because I'm afraid all the time of other people and I don't want to be… I guess it's my ideals of who I am; I'd rather be someone who's not afraid… so when I start freaking out about stupid things that probably aren't going to happen, like someone attacking me, that makes me feel sort of pathetic – Sophie

The paranoia experience often affected the adolescents’ self‐concept, with most describing negative self‐appraisals in relation to paranoia. This included appraisals about feeling persecuted, with paranoid thoughts triggering rumination about what makes them a target and also a sense of worthlessness. However, appraisals also often included self‐critical judgements about having paranoid thoughts. Nine participants described how unfounded suspicions made them feel “crazy”, “weird”, “stupid”, and, importantly, different from their peers. A few also described shame and guilt over mistrusting people without reason. The impact of paranoia on self‐concept left several adolescents with a desire to be different from how they were, whilst a number described paranoid behaviour becoming part of their identity. Paranoia could also prevent adolescents from expressing their personalities, experienced as a loss of self. This was notable for Emily who struggled to understand her mistrustfulness as she was sociable in the past, reflecting that: “it's not me.”

#### Held back from teenage life

I should be able to go out with my friends and enjoy my time, I shouldn't have to worry about being kidnapped or murdered or anything else, because I'm only 15, I'm still very young and I shouldn't have to grow up with these worries. I should be able to enjoy my life while I can. – Holly

The adolescents described how paranoia could hold them back from doing the things that they enjoyed and engaging with normal teenage life. This was often at odds with their expectations of how teenagers should be, with several describing sadness at feeling unable to go out and have fun like their peers. Multiple areas of daily life were affected, including concentration on schoolwork, struggling with everyday tasks, and an amplifying effect of paranoia on other difficulties (e.g., depression). However, the most prominent concern was a feeling of social restriction. Patterns of avoidance and withdrawal limited participants’ options for socialising, and several expressed sadness at spending most of their time indoors or alone. When adolescents did go out, intrusive paranoia and an attentional focus on threat typically interfered with their enjoyment, as described by Megan: “I'm focusing more on, ‘don't get beat up’, than actually having fun.” The social restrictions of paranoia often made it hard to fit in with peers, further contributing to adolescents’ views of themselves as outsiders. A number of participants also reflected on a conflict between paranoia and the pressure to fit in, struggling to weigh up the ‘social risk’ (Blakemore, 2018) of not going out against the risk of harm if they do. This included the possibility of being judged for their paranoia and, as a result, disguising it around friends:

I don’t want to tell too many people and they turn round and, [say] like, ‘you’re crazy; sorry, see you later, bye’… You've got to kind of disguise looking around funny at people and when you get the feeling there's someone out to get you, you've just got to put it past you. You've got to be like, if that happens, it happens, because if my friends find out, they're just going to tell me I'm crazy. *–* Katie

#### Disconnected from friends

[Paranoia] stopped me from talking to a lot of people that I'd like… I’m too scared to talk to them, I’ve always been too anxious, so it's stopped me from potentially making really good friendships… and a lot of my relationships, I've lost a lot of friends because I've distanced myself from them and they've just kind of given up and stopped talking to me, that happens a lot. – Lucy

An important consequence of paranoia was a sense of disconnection from friends, typically resulting in loneliness and isolation. For a number of adolescents, this was partly linked to feeling alone with paranoid concerns that others could not understand. However, most often it was due to mistrust making it hard to connect with others and form close friendships. This was pertinent for Nathan who described mistrust as a “big red cross [that] represents a barrier between me and everyone else.” Alongside giving little of themselves away, several participants described how quietness in social situations made them unapproachable or stopped them from initiating conversations with potential friends. Social avoidance also left adolescents with limited opportunity to meet new friends, and the tendency to decline invites and push people away made it hard to maintain friendships. Difficulties making and keeping close friends were often a source of sadness and frustration for adolescents, with several describing it as the most challenging part of paranoia.

#### Resigning and resisting

The final theme represented an ongoing tension for adolescents in their adjustment to paranoia between “reluctantly resigning” to the experience and “trying to resist” its impact.

##### Reluctantly resigning

I just sort of get on with life and I live, like, day by day as it is. I don't really understand everything about why it happens, but I’ve come to terms that it's not going to get any better, so I just have to live with it. – Nathan

Many adolescents described reluctantly resigning to paranoia due to a belief it could not be controlled, and, thus, had to be endured. There was a process of getting used to paranoia, with its first onset experienced as most worrying, before then becoming a more normal part of life. Alongside coming to terms with the paranoia experience, a number of participants also described acceptance of the social consequences, including isolation and a lack of close friendships. Reluctant resignation was most present for the six adolescents who described their paranoia as persistent, unchanging, or getting worse, and was typically associated with feelings of sadness and frustration. However, several other participants described an uncertain view of the future, involving partial acceptance that paranoia will always be there alongside hope that in some way it improves. A few participants also expressed weighing up the negative impact of paranoia against its protective function, leading to ambivalence about change:

It's kind of good to have these thoughts of worry because if something did happen you'd be aware, you'd be able to do something, but at the same time it does put you down – Holly

##### Trying to resist

I think I just kind of, not got used to it, but just realised what was happening. Like, maybe people aren't out to get me, but then again I've always got that slight suspicion maybe somebody is. But I think, you know, you've just kind of got to push forward and get on with it, because if not you're just going to be stuck, like panicking all the time and not leaving the house. – Katie

Despite the reluctant resignation that several adolescents described, most participants conveyed attempts to resist the experience and impact of paranoia. This included attempts to cope with paranoid thoughts, often through distraction or managing anxiety. A number of adolescents were also trying to let their guard down in order to rebuild trust in others, realising “I have to trust more people to get through life” (Holly), or trying to maintain a social life despite their fears. For several participants this was aided by re‐evaluating the likelihood of harm and reassuring themselves that they were safe. A few participants had started to challenge avoidance and face their fears, which, although difficult, helped them feel safer and learn that they could cope.

## DISCUSSION

This study set out to understand the experience of paranoia in help‐seeking adolescents with common mental health problems. All twelve adolescents interviewed had paranoid worries of physical harm from others, although concerns of deliberate social, emotional, and sexual harm were also described. This paranoia was often interconnected with other psychiatric difficulties, and almost half of adolescents viewed paranoia as central to the reasons they were seeking care. The clinical impact was clear: paranoia caused high distress, worsened other mental health problems, and was harmful to social relationships. Almost all participants said they wanted help for paranoia, yet none thought this had been offered by CAMHS. Paranoia may be an important issue that needs greater attention in adolescents presenting in clinical services.

A journey of paranoia emerged from the accounts. It started with the discovery of threat and vulnerability, led to a paranoid experience of mistrust and fear of others, and, finally, an adjustment to the impact of paranoia in daily life. This journey involved navigating multiple tensions, with adolescents trying to balance independence with vulnerability; trust with mistrust; and a desire to socialise with a fear of what people might do. But young people also knew that their paranoid concerns were excessive and, as a result, were still trying to make sense of them and decide how to respond. This reflected a key decision point for adolescents: reluctantly accept paranoia as a part of life or resist its impact and overcome it. The next stage of a young person's journey is likely to depend on the outcome of this decision.

The accounts support the cognitive conceptualisation of paranoia in adults as an inaccurate threat belief that activates anxiety‐related processes concerned with anticipating danger (Freeman, 2016). This was evident in the adolescents’ experience of paranoia, in which the anticipation of threat, overwhelming fear, and defensive strategies were central features. Further supporting the cognitive model, the onset of paranoia in participants was rooted in emerging beliefs about others as threatening and themselves as vulnerable (Freeman, 2016). For several adolescents, these beliefs and subsequent paranoia generalised from past experiences of objective (e.g. trauma/bullying) or subjective (e.g. anomalous perceptions) threat (Freeman et al., 2013, 2015; Shevlin et al., 2015). But the accounts also highlighted that, in adolescence, several normative changes in the social world may tilt some young people towards paranoia. These changes started in secondary school where challenging peer interactions typical of adolescence could provide new reasons to be mistrustful. This included incidents of betrayal within friendships that led to greater uncertainty about others and altered expectations of trust.

Social changes in adolescence, however, extend far beyond the peer group. A novel insight from this study was that increasing independence in adolescence – paired with a maturing awareness of danger – can result in a transitional state of vulnerability where young people are more exposed to potential harm but feel unprepared to manage the risks. Together, we propose this heightened vulnerability could result in an adaptive shift towards paranoia in adolescence, prompting young people to be more cautious as they start to navigate the dangers that exist in the world. For most adolescents, we expect this caution will lessen over time as they get older and more confident in their autonomy – consistent with evidence that rates of paranoia may decline with age (Freeman et al., 2011). If feelings of vulnerability persist, however, a worsening trajectory of paranoia throughout adolescence is likely to ensue.

### “So many girls are going through this” – The realities of sexual harm

An important insight from the accounts was that girls may feel especially vulnerable in adolescence as they discover the realities of sexual harm – potentially contributing, at least partly, to the higher rates of paranoia in girls at this age (Bird et al., 2019, 2021; Ronald et al., 2014).^1^ Sexual harm is a threat that, although it affects a minority of males, permeates the daily lives of women and girls. A recent survey of over 22,000 women found that before they were 18 years old, 80% had been sexually touched without consent and 33% had been forced or coerced to have penetrative sex (Taylor & Shrive, 2021). Our participants described how this reality is further emphasised to girls through the media, warnings from adults, and even their friends – surveys show that 30% of adolescent girls know other girls their age who have been sexually assaulted (Girlguiding, 2018). As highlighted by several participants, teenage girls are also made aware of their vulnerability to sexual harm through harassment by men in public, including ‘catcalling’ (i.e., wolf whistling and sexualised comments) and unwanted touching (Southgate & Russell, 2018). Street harassment is often targeted at young girls, with 85% of women having these experiences before the age of 17 (Hollaback, 2014). These experiences can erode girls’ sense of safety in public; indeed, 1 in 3 adolescent girls in the UK are reluctant to go out in their local area because they do not feel safe (The Children’s Society, 2017). Although it is undeniably important for girls to be mindful of risks, the persistent message that they are vulnerable and must be cautious is likely to create for some girls a worldview in which excessive mistrust thrives.

### Limitations

The study has several limitations. First, participants included more girls than boys – although this reflects higher rates of paranoia in girls within CAMHS (Bird et al., 2021). A deeper understanding of paranoia in adolescent boys will be required. Second, all participants were White British and lived in a relatively affluent area of the UK. Understanding experiences of paranoia in adolescents from different minority backgrounds is essential, especially those with greater adversity and social disadvantage which are risk factors for paranoia (Wickham et al., 2014). Finally, there was also a lack of service user involvement and diversity within the research team. Despite these limitations, the findings provide valuable first‐person insights and a developmental framework for conceptualising paranoia in adolescents as an understandable response to a changing and often threatening social world. The journey of paranoia, outlined in Figure 2, could be used by clinicians to frame discussions about paranoia with adolescents, supporting them to better understand their own journey, whilst also informing the future development of effective interventions.

## AUTHOR CONTRIBUTION

**Jessica C Bird:** Conceptualization (equal); Data curation (equal); Formal analysis (equal); Methodology (equal); Project administration (equal); Writing – original draft (equal). **Daniel Freeman:** Conceptualization (equal); Supervision (equal); Writing – review & editing (equal). **Felicity Waite:** Conceptualization (equal); Methodology (equal); Supervision (equal); Writing – review & editing (equal).

## Data Availability

The full dataset (i.e. whole interview transcripts) is not available due to ethical and privacy restrictions. Participants provided consent for the public use of anonymous quotes only, with an agreement that entire transcripts would not be shared beyond the research team.

## Footnotes

^1^
Quote from participant with pseudonym ‘Lucy’ (17 years).
