# Supplementary material for: The journey of adolescent paranoia: A qualitative study with patients attending child and adolescent mental health services
Source: Psychol Psychother. 2022 Feb 12;95(2):508–24. doi: 10.1111/papt.12385 (PMC9304248; doi:10.1111/papt.12385)
Supplement: Supplementary file 1 [file PAPT-95-508-s001.pdf]

The journey of adolescent paranoia: A qualitative study with patients attending child and adolescent mental health services.

SUPPLEMENTARY MATERIALS

1. Interview schedule development

The interview schedule was developed by all authors, with consultation from an external academic specialising in IPA. A primary consideration was ensuring the interview would facilitate young people's ability to talk freely about their experiences. As adolescent patients can often struggle to open up due to anxiety or self-consciousness, the interview schedule included more questions and possible prompts than a typical adult IPA study (Smith et al., 2009). Close attention was also given to ensure the language was clear and age appropriate. Importantly, the interview questions (and the study information provided in recruitment) did not refer to 'paranoia' due to the associated stigma of this word, with more relatable phrases such as 'mistrust' and 'worries about people' used instead.

The core interview questions reflected the study aim: exploring participants' understanding of the development, experience, and impact of paranoia. The interview started with the positively framed topic of trust to help adolescents feel at ease, before the more challenging content of paranoid concerns was explored. To consider the clinical context of participants' experiences, participants were also asked about whether paranoia was related to their other mental health problems and experiences of help-seeking.

The interview schedule was designed to be used flexibly to follow the young person's narrative, incorporate their own vocabulary, and provide verbal prompts to encourage elaboration (e.g. "can you tell me more?", "how do you mean?"). Particular attention was given to ensure the interview questions were consistent with IPA methodology in exploring how participants understand their experiences. Based on this, general prompts were used throughout to encourage reflection on the meaning of participants' responses to the questions (e.g. "what do you think about that?", "why is that?").

The interview was piloted with the first participant: although no changes to the content of the interview schedule were necessary, this interview highlighted the need to use more follow up prompts to encourage deeper reflection from participants on the meaning of experiences.

2. Interview schedule

General prompts used throughout

- Can you tell me a bit more about that?
- How come? / How do you mean? / In what ways?
- What do you think about that?
- What's that like?
- How do you make sense of that?
- Any more examples?

Part 1. Conceptualising trust

"As you know, this interview is about young people's experiences of feeling mistrustful. But to start off with it would be good to think about what trust is as this will be different for different people?"

1. Could you tell me what trust means to you?
   Potential prompts:
   - What does it make you think of?
   - Do any thoughts or pictures come to mind?
   - What does it feel like [to trust someone]?

2. How do you decide that you can trust someone?
   Potential prompts:
   - Any examples of people you trust?
   - What's it like when you do trust?

Part 2. Current experiences of paranoia

"When we last met you started to tell me about some of the times you feel mistrustful and worried about other people"

3. Can you tell me more about the worries you've been having?
   Potential prompts:
   - Can you give any examples? [refer to completed paranoia questionnaire if needed]
   - Thoughts / feelings in those moments
   - What happens in those moments / what do you do?

4. What is all of this like in day-to-day life?
   Possible prompts:
   - School / home / when out and about
   - Social media
   - Friendships / relationships?

5. How do [these worries] fit with how you see yourself?

6. What has been the most challenging part of [these worries]?

7. How do [these worries] link with the other problems you come to CAMHS for?
   Possible prompts:
   - [Refer to demographic sheet for listed presenting problems]
   - Similar or different? Connected or separate?

Part 3. Changes over time

8. "[Insert summary of paranoid concerns]. Could you tell me about any times the [worries] are better or worse?
   Possible prompts:
   - Why is that?
   - What influences how it changes?
   - Why do you think that is?
9. How did these [worries about people] first start?
   Possible prompts:
   - Was anything going on at that time?
10. How has it changed over time?

Part 4. Help-seeking

11. Have you spoken to anyone about these experiences?
    If yes: What was this like?
    - Experiences of professionals?
    - What has been helpful / unhelpful?

    If no: Why not?
    - What is it like to keep this to yourself?

Part 5: Ending questions

[Provide a brief summary of each section and ask if anything else want to add]

12. Is there anything we haven't talked about that you think is important to know?
13. Do you have any other questions for me?

Debrief, check-in, and feedback.

3. Demonstrating validity

Yardley's (2008) criteria for demonstrating credibility (i.e. validity) in qualitative research were used throughout the study. First, 'sensitivity to context' includes a sensitivity to relevant theory/research, the sociocultural setting, participant perspectives, and ethical issues in the study design and analysis. Second, 'commitment and rigour' includes thorough data collection, rigorous analysis with sufficient breadth and/or depth, methodological competence, and prolonged engagement with the topic. Third, 'coherence and transparency' refers to consistency between the chosen qualitative framework (e.g. IPA) and the methods used, the clarity of the findings, transparency in the methods and findings, and the use of reflexivity. Finally, impact and importance refers to a consideration of the practical, theoretical, and socio-cultural implications of the study. Details of how these four criteria were demonstrated in this study are shown in Table S1.

Table S1. Strategies undertaken to address Yardley (2008) criteria for ensuring validity in qualitative studies.

Criterion	Strategy employed
Sensitivity to context	• In-depth review of the literature on adolescent development, models of paranoia, and previous qualitative reports of paranoia.<br>• Consideration of the age and clinical context of participants in the study design to facilitate engagement and their ability to open up.<br>• Consideration of ethical issues in study design.<br>• Contextualisation of the analysis within participant demographics and the clinical context in which paranoia presented.<br>• Use of verbatim quotes to support claims.<br>• Use of reflexivity to consider influence of researcher on emerging analysis within supervision and a reflexive log.<br>• Bracketing interview prior to data collection
Commitment & rigour	• Prolonged engagement with the topic of adolescent paranoia.<br>• Purposive sampling of adolescents with experience of paranoia.<br>• Rigorous analytic process closely following recommended steps within IPA.<br>• Ensuring balanced representation of all 12 participants in analysis and selected quotes.<br>• Use of supervision to triangulate perspectives and perform credibility checks of emerging analysis.<br>• Independent rating of sections of transcript.
Transparency & coherence	• Detailed description of the method.<br>• Ensuring method and results consistent with IPA.<br>• Full interview schedule included with rationale for the content<br>• Audit trial of decision making and analytic process.<br>• Exploration of contradictions in findings.<br>• Regular supervision to develop coherent interpretation.<br>• Reflexive statement
Impact & importance	• Focus on clinical presentations of paranoia in a patient sample.<br>• Consideration of clinical implications of findings for CAMHS services and the treatment of paranoia in young people

3.1. Reflexivity

Reflexivity was used at every stage of the study to consider how the author's personal experiences, assumptions, and biases were influencing the findings. In the spirit of IPA, the aim of reflexivity was not to completely 'bracket' from these assumptions, but to curiously engage with them (LeVasseur, 2003). Prior to data collection a bracketing interview was conducted to reflect on these assumptions, although it was also recognised that one cannot fully predict how biases will arise until faced with the data (Smith et al., 2009). In the analysis, the author adopted a curious stance involving an assumption that they did not understand the participants' experiences and active reflection on their reactions to what participants said. These reflections were recorded in a reflexive journal alongside the analysis and elicited during reflexive conversations in supervision.

3.2. Supervision

Regular supervision was used throughout the analysis to triangulate perspectives, perform credibility checks, and collaboratively refine the emerging interpretation (Yardley, 2008). At the idiographic level, preliminary versions of each participant's theme structure were reviewed and refined in supervision with FW, alongside the annotated transcripts, to ensure they provided a credible and coherent narrative of the young person's experience. The theme structures therefore went through numerous iterations and were only finalised upon consensus agreement in supervision. Supervision with FW was further used to ensure the analysis adhered to the principles of IPA and was sufficiently interpretative, prompting a move beyond the descriptive to consider higher levels of interpretation (Smith, 2004). This was particularly important with early participants for whom the interpretations were generally more cautious. As a result, after the final interview had been analysed, the first three were re-analysed in full to produce stronger interpretative accounts that matched the depth of subsequent interviews.

The development of themes across participants was collaboratively refined in supervision with FW and DF, over seven one-hour meetings, to enrich the interpretation and ensure it was theoretically coherent. During these meetings, each member of the research team offered unique perspectives - both from individual areas of expertise and involvement at different stages of the analysis - that enhanced the credibility of the final account. To ensure transparency, an audit trail of the analytic process was maintained including a record of decision making and evolving versions of the idiographic and overarching analyses.

3.3. Independent rating

IPA recognises that different analysts bring unique interpretations to a transcript (Smith et al., 2009). Therefore, to further enhance the rigour of the analysis, a clinical psychologist external to the study independently analysed clean sections of transcript to the level of identifying emergent themes. In line with the principles of IPA, the objective of independent rating was not to assess inter-rater reliability. Instead, the goal was to consider the coherence of the emerging analysis and how it was being shaped by the author's particular perspective (Yardley, 2008). Despite this, comparison of the independent and original sections of analysis revealed a high level of consistency. The specific phrasing was often different, but the content of independently coded themes could be matched to those in the original analysis. No new themes were identified. Notably, the original analysis included greater detail and interpretation than the independent coding which was more descriptive. This reflected the author's commitment to the topic through prolonged engagement and familiarity with the research area and client group (Yardley, 2008).

4. Participant theme contributions

Table S2. Summary of participant contributions to each theme

Theme	Participant											
	1	2	3	4	5	6	7	8	9	10	11	12
**Discovering threat & vulnerability**												
Losing trust in peers		x		x	x	x	x	x	x	x	x	x
Growing into an unsafe world			x			x		x	x	x		x
Threatening experiences	x	x		x	x			x		x	x	x
Vulnerability to threat	x	x	x	x	x	x	x	x	x	x	x	x
▪ Uncertainty about others	x	x			x	x			x	x	x	x
▪ Self as a target	x	x	x		x		x	x			x	x
▪ Defenseless and alone	x	x	x	x		x	x	x	x	x	x	x
**The paranoia experience**												
Struggling to trust	x	x		x	x	x	x	x	x	x	x	x
Anticipating threat	x	x	x	x	x	x	x	x	x	x	x	x
Overcome with fear	x	x	x	x	x		x	x		x	x	x
Keeping safe	x	x	x	x	x	x	x	x	x	x	x	x
**Making sense of paranoia**												
Confused logic	x	x	x	x	x	x	x	x	x		x	x
Impact on self-concept	x	x	x	x	x		x	x	x		x	x
Held back from teenage life	x	x		x	x	x	x	x	x	x	x	x
Disconnected from friends	x			x	x	x	x	x	x		x	x
Reluctantly resigning	x	x	x	x	x	x	x	x	x		x	x
Trying to resist	x	x	x	x		x	x	x	x	x	x	

Note. 1: Katie, 2: Megan, 3: Jack, 4: Ashley, 5: Nathan, 6: Chloe, 7: Emily, 8: Sophie, 9: Holly, 10: Sam, 11: Olivia, 12: Lucy

5. Additional quotes

Table S3. Additional quotes for the first level of superordinate theme "discovering vulnerability and threat."

Discovering vulnerability and threat	
**Losing trust in friends**	
Chloe, 14	"I guess it's really easy to break trust, because I've told people things before and they've gone behind my back and told other people things. . . After your trust has been broken once, you don't really know how to, like if you should talk to anyone else, as you don't know what people are like?"
Olivia, 16	"When I was younger I used to sort of, tumble into people quite easily. I'd sort of go, oh, do you want to be friends, so talking to them loads and I'd tell people things and then that sort of backfired… but now I've had experiences with people and I've had that prior knowledge of what people are like, I tend to be more wary"
**Growing into an unsafe world**	
Sophie, 16	"It was a shock, so I'd start hearing on the news about all the different things that were happening... because I didn't hear much about it when I was a child, when I started secondary school and I was introduced to it a lot more, it was something I was suddenly aware of and because of this I was sort of like, okay, everyone's telling me I need to be careful, so I need to be really careful because it's probably something that's going to happen"
Holly, 15	"I've always been aware of what can happen with strangers, or anyone, it doesn't need to be strangers, it can be someone you trust dearly and they can still do horrible things to you… And I think, as I've got older and matured, and with social media where you hear everything on the news and everything gets passed round, you hear about it so much more and it's just like, this seems to be happening a lot, I'm not going outside because if there's everything happening a lot more, why should I risk myself? I can just stay home."
**Threatening experiences**	
Lucy, 17	"[The paranoia] kind of started when I was about 14-15, because that's when the comments started being given to me and people started honking their horn and whistling, that's when I started to feel like unsafe outside, and then I got mugged and then this weird guy started talking to me as well."
Ashley, 12	"There was someone in a black coat, all black, behind me, and then next thing I looked back, he had disappeared, which, it was quite creepy… When I saw that man in black, I think that's when it completely started, that's when I, that's when people followed me… that's when I started to mainly think about it."

Table S4. Additional example quotes for the second layer of superordinate theme "discovering threat and vulnerability", representing the three subthemes of "vulnerable to threat."

**Vulnerable to threat**	
**Uncertainty about others**	
Holly, 15	"You don't know what people are capable of, and that's very scary and kind of links to don't judge a book by its cover, because someone could look really petite, innocent, really kind, but really they could be very strong and they could have a record or something, you don't know. So that also leads to like, not trusting very well because obviously looks can be deceiving"
Sam, 12	"But no matter what, you can't be too safe… Even with your friends, you can't trust them sometimes, because still they're your friends, you think you can trust them, but they still can spread the words… so you can pretty much never be too safe because people can double-cross you or triple-cross you and you just can't trust people that you don't know, even your friends."
**Self as a target**	
Nathan, 15	"I think a lot of people despise me, and if lots of people despise me, a couple of people are probably going to do something about it."
Megan, 16	"Because everyone says I'm a pretty girl… Sometimes it makes me feel dirty, because all the men come up to me, and I feel like I look like a slag and stuff."
Jack, 11	"The way I look or, the way I look… I just think that I look weird and people might, like, talk about me or something."
**Defenceless and unprotected**	
Sophie, 16	"I used to be confident that I'd be able to defend myself, and then I guess I got, when I started feeling less confident about myself, I also was like, well, I'm pathetic, I'm not going to be able to protect myself from anything, so yeah, I got more anxious about what other people could do and say."
Ashley, 12	"I think also what I feel like I'm going to get kidnapped or someone's following me, I also think it's because, like, I might be, like, left, I might be abandoned. I think it's because if I ever do, which I might not but it's like, you never know, that my parents would never look for me or anything."

Table S5. Additional example quotes for superordinate themes of "the paranoia experience."

**The paranoia experience**	
**Struggling to trust**	
Megan, 16	"I think trust is a bigger thing [to me] than a lot of other people my age… Probably because a lot of bad things have happened to me, so I just think it's like the one thing that I've got, like, left… I take it really seriously."
Lucy, 17	"If I meet someone new and I become friends with them, immediately I'm like, 'oh my gosh, this is someone that likes me and they're friends with me', so I trust them straightaway… then my brain's like, 'oh, but literally everybody in your life before has messed with your trust', so I start to doubt myself and I stop telling them things and I cut myself off from them. So it's kind of, because I'm so desperate to trust someone, I let it happen really quickly, then I over-think it all and stop myself."
**Anticipating threat**	
Olivia, 16	"When I'm walking I usually look behind me a lot, I tend to look straight at the ground, I don't like looking at people just in case they'll do something… It sort of feels like my brain makes me listen to every conversation that everyone is having, so it's like I'm getting an overload of sound and it's like a sensory overload."
Emily, 16	I take notice of their body language and just little things they say, I think maybe that's a sign that someone's going to be, like, I don't know, mean to me or, I don't know. It's really weird.
**Overcome with fear**	
Jack, 11	"Sometimes I can take my mind off of it, if my Mum, like, speaks with me, but then sometimes it just doesn't work. Sometimes I get, like, proper anxious, I try and breathe, but I just get, I just get so anxious of people staring at me."
Ashley, 12	"When I get anxious, it's not just anxious, it's, like, angry at the same time… So like I said, I get scared, so I feel so unsafe when I don't have my phone, and my Mum was trying to take my phone for the night, because I hadn't cleaned up something… so when she tried to take my phone I literally had this big breakdown, my Mum had to call [crisis team]."
**Keeping safe**	
Sophie, 16	"I'll be a lot more cautious about who's around me and where I am and trying to fit in to not draw attention to myself. And I'll try and get out of the situation as quickly as possible"
Holly, 15	"If I'm in a room, I always try and identify everything in the room, because if something bad happened, I would be able to make a plan with that. So when I'm out in a public place, I'm always kind of ready for anything. Like if I'm standing still, I always stand in a stance where if someone tried to push me over, they wouldn't be able to do it very easily."

Table S6. Additional example quotes for superordinate theme “adjusting to paranoia.”

**Adjusting to paranoia**	
**Conflicting logic**	
Lucy, 17	“I'm convinced they're following me, and I'm like, oh my god oh my god. So I always go into different shops to try and get away from them, but no-one is ever following me, like no-one, nothing ever happens to me. I'm just really convinced that people do, I don't know, it's kind of like a delusion that I have”
Jack, 11	“It's never happened before. But at the time, I say it now, but at the time when someone is staring at me, I kind of just forget about everything that I've said before… but nothing's ever happened, that someone's, like, a stranger's hurt me or anything.”
**Impact on self-concept**	
Nathan, 15	“I just want to be like everyone else, and [paranoia] stops me from being like everyone else… I just want to be sort of a functioning member of society; one that is liked, and one that's understood, but that's not going to be happening any time soon… because these thoughts aren't going away.”
Katie, 16	“I kind of see myself as crazy. Like, everyone else just goes out and has fun, and then I go out and I'm all panicky and I’m like ‘oh, there's some randomer there’, or ‘this is happening’, so I do kind of see myself as, you know, a bit loony.”
**Held back from teenage life**	
Ashley, 12	“I'm just in my room; I don't even open my curtains, I don't even know what light is any more. Which is annoying, because I'm a really out-doors-ey person… I hardly ever leave my room anymore.”
Emily, 16	“It's not really that nice because I can see people on social media going out to their friends' parties and things like that, or just spending time together and I just don't have trust in people to do that… I get really sad at home, and I'm like, oh, I want to get out, but when I do I just get really panicky so I just don't bother.”
**Disconnected from friends**	
Chloe, 14	“[The most challenging part is] lack of friends. Because I had a best friend, and then we just drifted apart. Sometimes it can feel really lonely. I just can't be close to people.”
Sophie, 16	“My old friendship group, they sort of, I don't see them much, but if I do see them, they'll be like, ‘oh my God she's here’, and joke about it. They've sort of gotten used to the fact that I'm not around anymore.”
**Resigning and resisting**	
Reluctantly resigning	
Katie, 16	“There's nothing you can really do to stop it, if that makes sense. You've just got to kind of get used to it.”
Emily, 16	“I still struggle with trusting people. I think I kind of always will now. I don't think it'll change because obviously you can't go back and change things, so that's probably going to stick with me forever really now.”
Trying to resist	
Holly, 15	“Sometimes I just refuse. I'll just sit there and in my head I'll just go, ‘I'm not going to do it, I'm not going to ruin my own time.’ Yeah, there are still worries and I'll occasionally look around and be very skitty, but sometimes if I'm out with the family or a family meal, I'm just like, I'm not going to ruin this because I don't know if this will happen again”
Sam 12	“You just try and persevere sometimes. So pretty much just, confidence is when you get a bit braver. So being brave is pretty much just saying, like, I'm not scared of you.”

References

LeVasseur, J. J. (2003). The problem of bracketing in phenomenology. *Qualitative Health Research*, 13(3), 408–420. 10.1177/1049732302250337

Smith, J. A. (2004). Reflecting on the development of interpretative phenomenological analysis and its contribution to qualitative research in psychology. *Qualitative Research in Psychology*, 1(1), 39–54.

Smith, J. A., Flowers, P., & Larkin, M. (2009). Interpretative phenomenological analysis: Theory, method, and research. London, UK: Sage.

Yardley, L. (2008). Demonstrating validity in qualitative psychology. In J. A. Smith (Ed.) *Qualitative psychology: A practical guide to research methods* (2nd ed., pp. 53–80). London, UK: Sage.
